# Topographic distribution of choriocapillaris flow deficits in healthy eyes

**DOI:** 10.1371/journal.pone.0207638

**Published:** 2018-11-15

**Authors:** Marco Nassisi, Elmira Baghdasaryan, Tudor Tepelus, Samuel Asanad, Enrico Borrelli, Srinivas R. Sadda

**Affiliations:** 1 Doheny Image Reading Center, Doheny Eye Institute, Los Angeles, California, United States of America; 2 Department of Ophthalmology, David Geffen School of Medicine at UCLA, Los Angeles, California, United States of America; 3 Ophthalmology Clinic, Department of Medicine and Science of Ageing, University G. D'Annunzio Chieti-Pescara, Chieti, Italy; Massachusetts Eye & Ear Infirmary, Harvard Medical School, UNITED STATES

## Abstract

**Purpose:**

To evaluate the topographic distribution of the choriocapillaris (CC) flow deficits in a population of healthy subjects.

**Methods:**

Using a swept-source optical-coherence tomography angiography (SS-OCTA) device, two repeated volume 6 x 6 mm and 3 x 3 mm scans were acquired in healthy subjects at the Doheny—UCLA Eye Centers. The en-face CC angiogram was binarized and analyzed for percentage of flow deficits (FD%) using a grid of progressive, concentric rings covering a circular area with a diameter of 2.5 mm (in the 3 x 3 mm scans) and 5 mm (in the 6 x 6 mm scans). The FD% for each ring was plotted against the distance from the fovea. The linear trendline of the resulting curve was analyzed and the slope (*m*) and intercept (*q*) were computed.

**Results:**

Seventy-five eyes of 75 subjects were enrolled and divided into three subgroups based on age (year ranges: 21–40, 41–60 and 61–80). For the entire cohort and within each subgroup, there was a significant association between distance from the fovea and FD% in both 3X3 mm and 6X6 mm scans, with flow deficits increasing with closer proximity to the foveal center. Age was a significant predictor for both *m* and *q* for both scan patterns, with older subjects showing a steeper slope.

**Conclusions:**

In SS-OCTA images, the topographic distribution of CC flow deficits varies with distance from the fovea and age. In particular, the FD% tends to decrease from the fovea towards the periphery, with a steeper decline with advancing age. These normal trends may need to be accounted for in future studies of the CC in disease.

## Introduction

The development of optical coherence tomography angiography (OCTA) has provided new insights into the retinal and choroidal microvasculature. allowing individual vascular plexi to be evaluated at extremely high resolution [1]. OCTA has thus opened the door for a precise correlation between vascular, structural and functional alterations *in vivo* in both a cross-sectional and longitudinal fashion.

Visualization and analysis of the choriocapillaris (CC) in particular, has been a topic of great interest with OCTA. The choriocapillaris represents an approximately 10-μm-thin layer of capillaries interconnected in a densely packed arrangement with very small intercapillary pillars located between the medium/large choroidal vessels and Bruch’s membrane [2]. Changes in CC are known to occur physiologically with increasing age and to be associated with a wide range of retinal diseases including age-related macular degeneration (AMD) and central serous chorioretinopathy (CSC), which are major causes of vision loss [3–6].

Imaging the CC *in vivo* is difficult because of the light scattering caused by overlying structures, in particular the retinal pigment epithelium (RPE)[7]. The introduction of swept-source (SS) OCTA has allowed a more reliable visualization of the CC thanks to greater sensitivity and the use of a longer wavelength which better penetrates deeper tissues and is less scattered by the RPE [8–11]. The high quality images provided by SS-OCTA can produce visualization of the CC which can resemble histology in many respects [10–12]. With typical processing approaches, OCTA images of the CC appear as grainy pictures where white pixels represents flow and black pixels represents areas of signal deficits where the flow is supposed to be absent or below the detecting threshold. A post-processing analysis of these images allows the quantification of the black pixels (flow deficits) within the CC. This type of analysis has been used in many studies as an indication of CC impairment in various retinal diseases [13]. Recently Spaide et al. measured the size and number of the flow deficits in subjects at different ages and in different diseases, and observed that these parameters are dependent on age and hypertension [5]. In previous studies, we have shown that choroid, as assessed by choroidal thickness, can show significant regional variation in normal individuals [14]. Thus, it is reasonable to hypothesize that the CC could also demonstrate significant regional variation (personal communication, Phil Rosenfeld). This hypothesis is supported by histologic studies which have demonstrated differences in the CC morphology in central compared to more peripheral regions [2]. These topographical variations in the CC may be significant confounders of studies which compare CC flow deficits within regions of interest in the macula [3,4,15,16]. To study the potential impact of these regional and age-related variations, we studied the topographical distribution of CC flow deficits in a cohort of normal subjects.

## Methods

SS-OCTA images from healthy volunteers were obtained as part of a normative study conducted at the Doheny-UCLA Eye Centers between December 2017 and May 2018. Eligible subjects were healthy, with no systemic or eye-related conditions, and confirmed by examination. Any refractive error greater than 3 diopters and presence of significant media opacities which could impact the quality of the OCT images were exclusion criteria for this study. The study was advertised at the Doheny-UCLA Eye Centers to all staff members, visitors and family to participate and volunteers, if eligible, were consecutively recruited.

The study was performed in accordance with the Health Insurance Portability and Accountability Act and adhered to the principles of the Declaration of Helsinki. All subjects provided written informed consent to participate in this observational study. The informed consent form and research was approved by the institutional review board (IRB) of the University of California–Los Angeles (UCLA).

All participants underwent a complete ophthalmic examination, including best-corrected visual acuity (BCVA) using Early Treatment Diabetic Retinopathy Study (ETDRS) charts, slit lamp biomicroscopy, tonometry and SS-OCTA.

### Imaging

Subjects underwent SS-OCTA imaging with the PLEX Elite 9000 device (Carl Zeiss Meditec Inc., Dublin, CA, USA) which uses a swept laser source with a central wavelength of 1050 nm (1000–1100 nm full bandwidth) and operates at 100,000 A-scans per second. This instrument employs a full-width at half-maximum axial resolution of approximately 5 μm in tissue, and a lateral resolution at the retinal surface estimated at approximately 14 μm. OCTA imaging of the macula was performed using two scan patterns: a 3 X 3 mm (300 A-scans x 300 B-scans) and a 6 X 6 mm (500 A-scans x 500 B-scans) scans centered on the fovea. The right eye of each subject was repeatedly imaged after pupil dilation to obtain two OCTA volume scan sets per area investigated with sufficient image quality (signal strength index (SSI)>7) that fulfilled the acceptance criteria of the Doheny Image Reading Center (DIRC), as previously reported [4,17].

The manufacturer’s fully-automated retinal layer segmentation algorithm was applied to the three-dimensional structural OCT data, in order to segment the CC slab as defined previously (10 μm thick starting 31 μm posterior to the RPE reference) [5]. This segmentation was then applied to OCTA flow intensity data to obtain vascular images. Maximum projection analyses of the flow intensity were performed to generate the *en-face* images of the CC plexus.

### Quantitative image analysis

The CC *en-face* image was exported and analyzed using ImageJ software version 1.50 (National Institutes of Health, Bethesda, MD; available at http://rsb.info.nih.gov/ij/index.html) [18]. The CC en-face images were binarized for quantitative image analysis of the signal deficits using the Phansalkar method (radius, 15 pixels) as previously described [5,19,20]. The CC *en face* images were then processed with the ‘Analyze Particles’ command (size: 0-infinity, circularity 0–1) in order to count the flow deficits as a percentage of each analyzed area (FD%).

**Fig 1:**
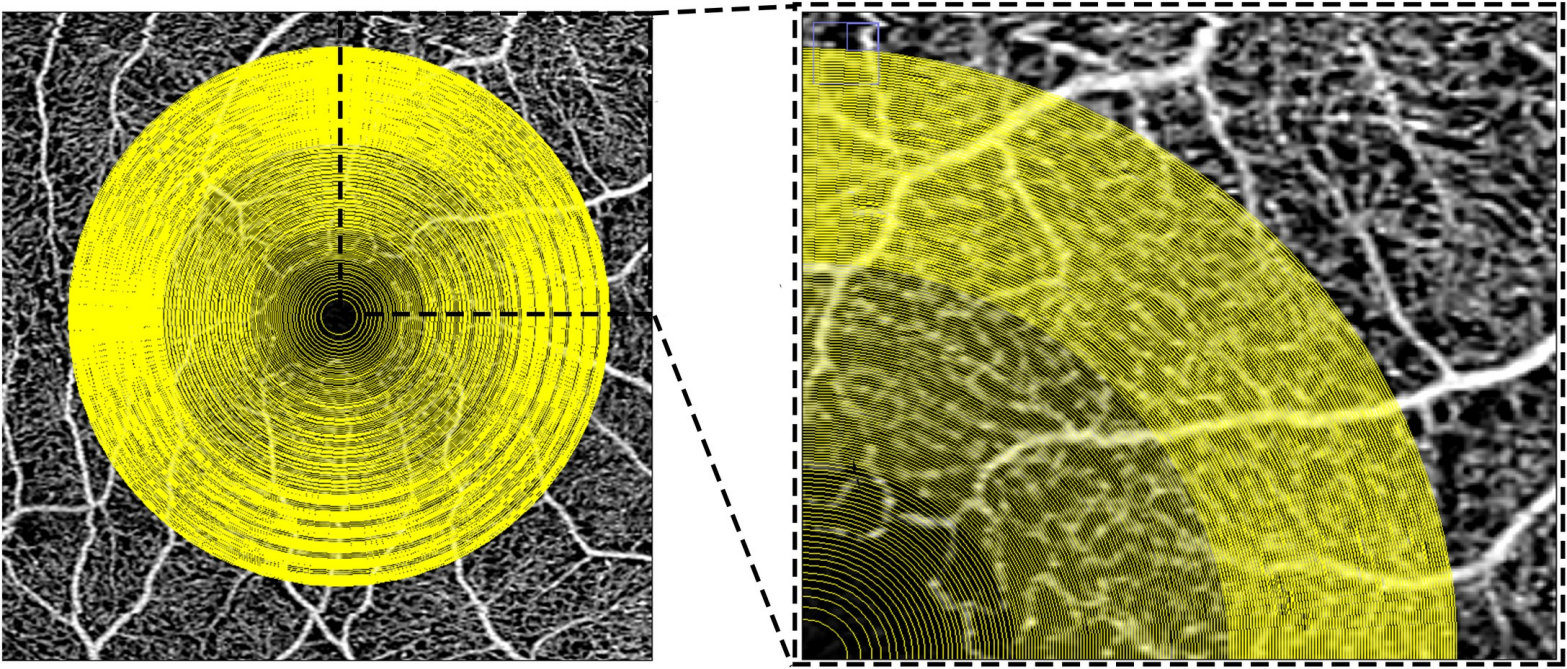
Multiple concentric rings grid used to analyze the choriocapillaris images in the study. Optical coherence tomography *en-face* 3X3 mm angiogram of the superficial plexus with the superimposition of the grid used for the analysis of the choriocapillaris in this study. The superficial angiogram was used as a reference to manually center the grid on the center of the foveal avascular zone. The box on the right (inset of the image on the left), shows a magnified view of the superonasal quadrant of the grid. The concentric rings of the grid are defined by progressively wider concentric circles starting from the fovea and ending at a diameter of 2.5 mm (in the 3X3 scans) and 5 mm (in the 6X6 scans). Note, the spacing between circles decreases with greater distance from the fovea in order to maintain more similar areas between the various concentric rings.

The analysis of the flow deficits was performed within a circular area with a diameter of 2.5 mm and 5 mm for the 3 X 3 mm and 6 X 6 mm, respectively. This roughly correspond to a circle measuring 854 pixels in diameter (note as the OCTA images were exported at a similar resolution, both the 3x3mm and 6x6mm *en face* OCTA CC images were composed of a similar number of pixels: 1024x1024). Starting from this outermost boundary, we constructed a grid of continuous concentric rings of progressively increasing width towards the fovea following these steps: (1) after delineating the outermost circle (radius: 427 pixel), we delineated an inner circle with a radius of 426 pixel, concentric to the first one and measured the area of the resulting ring (2678 pixel^2^) as:

$$Ring\;Area=\pi \times (r_{2}^{2}-r_{1}^{2})$$ (A)

where r_2_ is the radius of the outer circle and r_1_ is the radius of the inner circle; (2) we progressively added inner concentric circles using the formula (A) such that the area of the resultant rings were within ±35% of the area of the outermost ring (2678 pixel^2^). It was necessary to increase the width of the more central rings in this way in order to maintain a somewhat similar area for the various concentric rings. If such an adjustment was not introduced and the ring width was instead maintained constant at all eccentricities, the innermost ring would only be composed of a few pixels which would reduce the reliability of subsequent flow deficits calculations. At the same time, we chose not to make all concentric rings of exactly the same area as the most central rings would be very wide and limit our ability to study differences in CC flow deficits as a function of distance from the foveal center. Using this “compromise” strategy, the size (area) of the rings varied from 1741 and 3615 pixel^2^ (i.e. 2678 ± 35%) (Fig 1).

The precise 35% threshold itself was arbitrarily chosen because it ensures the minimum possible fluctuation in ring areas. This can be explained through an example: with the choice of a threshold of 30% from the outermost ring (range of 1875–3482 pixel^2^), the delineation of progressively smaller concentric circles leads to the 1-pixel wide ring of 1880 pixel^2^. The subsequent 1-pixel ring would be under the 30% threshold; hence, we would have to widen the ring to 2-pixels. In this case, the resultant ring has an area of 3742 pixel^2^ which is over the upper limit of the chosen range. On the other hand with a threshold of 35% (range: 1741–3615 pixel^2^), when we reach the 1742 pixel^2^ ring, the widening of the subsequent ring to 2-pixels, results in an area of 3466 pixel^2^, which is still within the 35% range.

For each image we used the *en face* angiogram of the superficial plexus to identify the fovea and center the grid (Fig 1).

All measurements were repeated by the same OCT grader (MN) on two different acquisitions in order to explore the “inter-scan” repeatability. Then, in order to rule out the possibility that the manual positioning of the grid on the fovea could affect the “inter-operator” repeatability, a second grader (EB) performed the analysis on one of the two acquisitions for each scan patterns.

### Statistics

**Fig 2:**
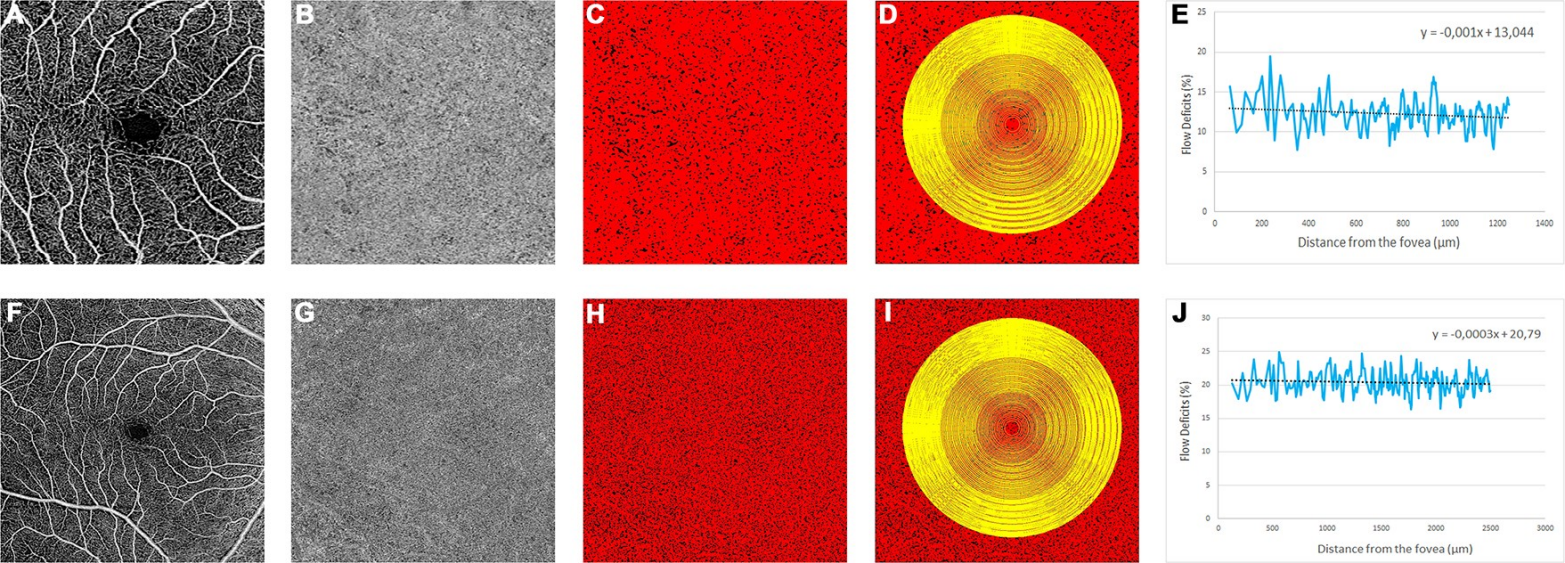
Example of flow deficits analysis with the two scan protocols used in the study. The two rows of images represent two optical coherence tomography angiography acquisitions (3X3 mm pattern in the top row and 6x6 mm patter in the bottom row) of the same subject. The angiogram of the superficial plexus (A and F) was used as a reference to center the grid in the center of the foveal avascular zone. The choriocapillaris (CC) angiogram (B and G) was automatically thresholded in order to obtain a binarized image (C and H) where the black pixels correspond to the CC flow deficits which were calculated superimposing the grid to the image. The percentage of flow deficits (FD%) was plotted versus the distance from the foveal center in order to obtain the resultant plots (E, J). The linear trendline (black line in E, J) of the curves were generated and the slopes and the intercepts of the corresponding equation were computed for further analysis.

For each image we plotted the FD% (y-axis) with the distance from the fovea (x-axis) on Microsoft Excel 2013 software (Microsoft Corporation, Redmond, WA). We then obtained the linear trend line of the curve and reported the slope (*m*) and the intercept (*q*) from its equation (Fig 2).

Statistical analyses were performed using SPSS Statistics version 20 (IBM, Armonk, NY). An ANOVA test and an unpaired t-test were performed to investigate the differences in slopes and intercepts between the age groups and between genders, respectively. A linear regression analysis was used to investigate: (1) the association between distance from the fovea and FD% overall and within pre-specified age groups; (2) the association between age, sex and the slopes and the intercepts. Inter-scan and inter-operator repeatabilities were assessed by calculating the intraclass correlation coefficients (± 95% confidence interval [CI]) for *m* and *q*.

All data are presented as mean ± standard deviation. A p value ≤ 0.05 was considered to be statistically significant.

## Results

A total of 75 eyes from 75 healthy subjects were enrolled in this study (mean age: 50 ± 17.15 years, range 23–80; 37 Females).

Overall, there was a significant association between distance from the fovea and FD% in both 3X3 mm and 6X6 mm scans as determined by the linear regression analysis. The regression coefficient was -0.08 (standard error (SE): 0.003) for an increase of 50 μm in distance from the foveal center in the 3x3 mm and -0.052 (SE: 0.003) for an increase of 100 μm distance from the foveal center in the 6X6 mm (Table 1).

**Table 1:** Univariate linear regression analysis of distance from the fovea versus percentage of flow deficits, for each optical coherence tomography angiography scan pattern.

	Percentage of Flow Deficits											
	Overall			Ages 21–40			Ages 41–60			Ages 61–80		
	Unstandardized coefficient (standard error)	Standardized ß Coefficient	P value	Unstandardized coefficient (standard error)	Standardized ß Coefficient	P value	Unstandardized coefficient (standard error)	Standardized ß Coefficient	P value	Unstandardized coefficient (standard error)	Standardized ß Coefficient	P value
Distance from the fovea (50μm increase) (3X3 mm scan)	-0.08 (0.003)	-0.872	<0.001	-0.016 (0.004)	-0.264	<0.001	-0.081 (0.004)	-0.741	<0.001	-0.143 (0.005)	-0.880	<0.001
Distance from the fovea (100μm increase) (6X6 mm scan)	-0.052 (0.003)	-0.765	<0.001	-0.018 (0.004)	-0.282	<0.001	-0.058 (0.004)	-0.686	<0.001	-0.08 (0.005)	-0.709	<0.001

The linear regression analysis was performed using the entire cohort, and for the three age-based subgroups. In all situations, there is a significant association between the distance from the fovea and the percentage of flow deficits (FD%).

Age was a significant predictor for both *m* and *q* in each scan patterns while gender was not associated with either (Table 2). An unpaired t-test between genders revealed no significant differences for *m* and *q* in both 3X3 and 6X6 mm scan patterns (Table 3).

**Table 2:** Multivariate linear regression analysis between age and gender and the slopes (*m*) and intercepts (*q*) of trendlines of the curves plotted for percentage of flow deficits versus the distance from the fovea.

	3X3 mm				6X6 mm			
	Slope (*m*)		Intercept (*q*)		Slope (*m*)		Intercept (*q*)	
	Standardized ß Coefficient	P value	Standardized ß Coefficient	P value	Standardized ß Coefficient	P value	Standardized ß Coefficient	P value
Age	-0.450	<0.001	0.409	<0.001	-0.430	<0.001	0.346	0.002
Gender	0.165	0.115	-0.061	0.570	0.142	0.181	0.003	0.982

In this multivariate linear regression analysis, age is the only significant predictor for both *m* and *q* for both optical coherence tomography angiography scan patterns.

**Table 3:** Mean slopes and intercepts of the linear trendlines of the curves obtained by plotting the percentage of flow deficits versus the distance from the fovea.

	3x3 SCAN								6x6 SCAN							
	Overall	Gender			Age range				Overall	Gender			Age range			
		Males	Females	p-value*	21–40	41–60	61–80	p-value**		Males	Females	p-value*	21–40	41–60	61–80	p-value**
Slope (*m*)	-0.0017 (0.0024)	-0.0021 (0.0024)	-0.0012 (0.0024)	0.138	-0.0003 (0.0019)	-0.0016 (0.0024)	-0.003 (0.002)	<0.001	-0.0005 (0.00069)	-0.00071 (0.0006)	-0.0004 (0.0006)	0.201	-0.0002 (0.0004)	-0.0006 (0.0009)	-0.0009 (0.0004)	0.001
Intercept (*q*)	13.68 (4.08)	13.96 (4.404)	13.4 (3.76)	0.559	11.63 (3.67)	13.66 (3.85)	15.77 (3.76)	0.001	20.66 (2.76)	20.67 (3.41)	20.66 (1.91)	0.987	19.55 (1.57)	20.7 (3.65)	21.89 (2.3)	0.011

Data are presented separately for the two optical coherence tomography angiography scan patterns (3X3 and 6X6 mm) and for the entire population and subgroups divided by gender and age range. All data are presented as means (standard deviation). P-value*: unpaired t-test; p-value**: one-way ANOVA.

### Age groups

We divided the population in three pre-specified sub-groups based on the age range: (1) 21–40 years (25 subjects, 12 Females); (2) 41–60 years (25 subjects, 13 Females); (3) 61–80 years (25 subjects, 12 Females).

**Fig 3:**
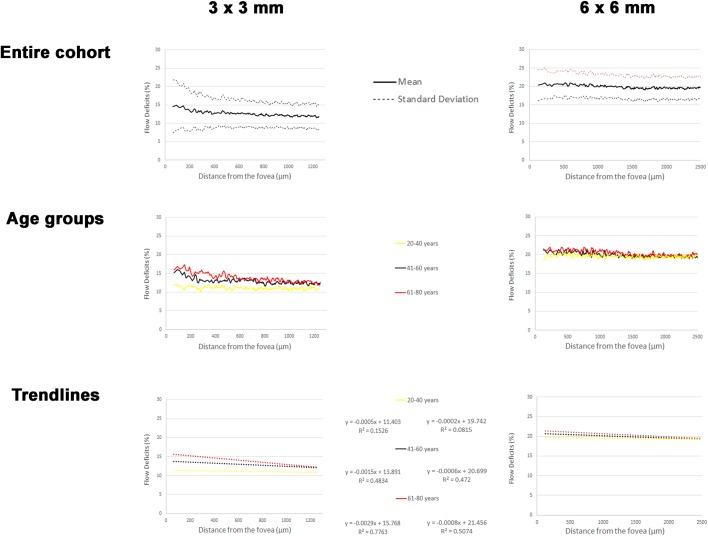
Graphical representations of the results of the study. Mean flow deficits percentage (FD%) plotted against the distance from the fovea for two optical coherence tomography angiography (OCTA) acquisition protocols (3 x 3 m and 6 x 6 mm). When divided by different age groups, the linear trendlines of the respective curves appear to have different intercepts and slopes; in particular, the 61–80 years group has a higher intercept and a lower slope meaning that in this group the FD% tends to be higher overall, and even more so as one approaches the foveal center.

Plots of the mean FD% versus the corresponding distance from the foveal center for the overall population and for each age groups are shown in Fig 3.

In the 3X3 mm scan there was a significant association between distance from the fovea and FD% in all three groups. For an increase of 50 μm from the fovea, the regression coefficients were -0.016 (SE: 0.004), -0.081 (SE: 0.004) and -0.143 (SE: 0.005) for the groups between 21–40 years, 41–60 years and 61–80 years of age, respectively (all p<0.001) (Table 1).

The mean *m* in the youngest group was -0.0003 ± 0.0019, while in the group between 41–60 years it was -0.0016 ± 0.0024 and in the oldest group *m* it was -0.003 ± 0.002 (one-way ANOVA (F(2,74) = 9.823, p<0.001) (Table 3).

The mean *q* in the youngest group was 11.63 ± 3.67, while in the group between 41–60 years it was 13.66 ± 3.85 and in the oldest group the *q* was 15.77 ± 3.76 (one-way ANOVA (F(2,74) = 7.544, p = 0.001) (Table 3).

In the 6X6 mm scan there was a significant association between distance from the fovea and FD% in all three groups. For an increase of 100 μm from the fovea, the regression coefficients were -0.018 (SE: 0.004), -0.058 (SE: 0.004) and -0.08 (SE: 0.005) for the groups between 21–40 years, 41–60 years and 61–80 years of age, respectively (all p<0.001) (Table 1).

The mean *m* in the youngest group was -0.0002 ± 0.0004, while in the group between 41–60 years it was -0.0006 ± 0.0009, and in the oldest group *m* was -0.0009 ± 0.0004 (one-way ANOVA (F(2,74) = 7.748, p = 0.001) (Table 3).

The mean *q* in the youngest group was 19.55 ± 1.57, while in the group between 41–60 years it was 20.7 ± 3.65 and in the oldest group the *q* was 21.89 ± 2.3 (one-way ANOVA (F(2,74) = 4.848, p = 0.011) (Table 3).

### Repeatability

For 3 X 3 mm acquisitions, the inter-scan repeatability demonstrated an ICC of 0.858 (95% CI 0.810–0.914) for *m* and 0.863 (95% CI 0.847–0.912) for *q*. The inter-operator repeatability was 0.991 (95% CI 0.976–0.996) and 0.997 (95% CI 0.996–0.999) for *m* and *q* respectively.

For 6 X 6 mm scans, the inter-scan repeatability had an ICC of 0.831 (95% CI 0.801–0.856) for *m* and 0.814 (95% CI 0.784–0.865) for *q*. The inter-operator repeatability was 0.990 (95% CI 0.975–0.996) and 0.999 (95% CI 0.996–0.999) for *m* and *q* respectively.

## Discussion

**Fig 4:**
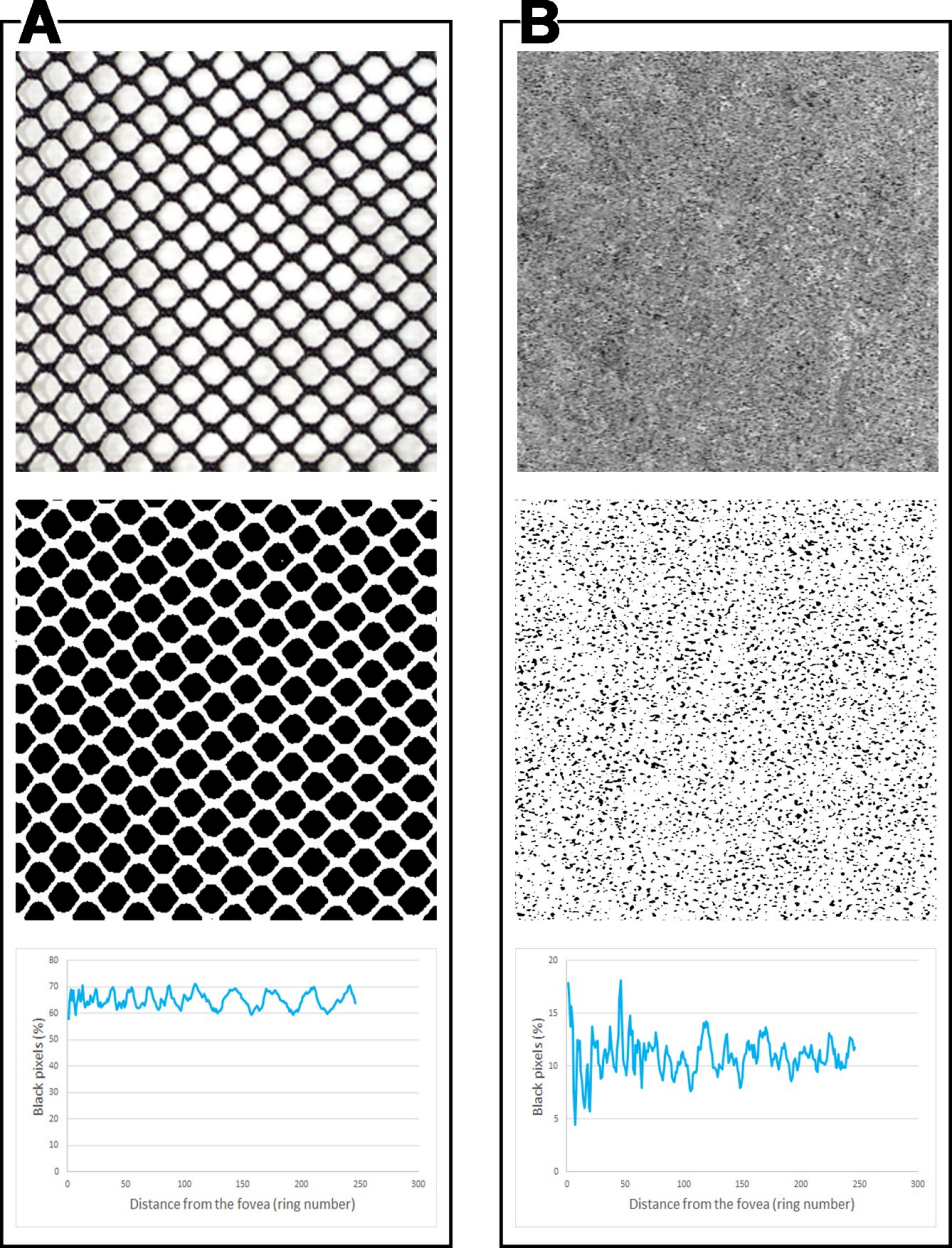
Multiple concentric rings analysis applied to a regular meshwork and a choriocapillaris angiogram. Application of the multiple concentric rings analysis method used in this study to two different images (first row): (A) a regular meshwork, (B) optical coherence tomography angiography (OCTA) image of the choriocapillaris (CC). After binarization (second row) and application of the analysis method utilized in this study, the shapes of the curves yielded by plotting the percentage of black pixels (y-axis) versus the distance from the center (x-axis) show some similarities. As expected, the more irregular CC meshwork yields a more irregular curve.

In this study, we developed a strategy to analyze the topographical distribution of flow deficits in OCTA *en face* images of the choriocapillaris in a continuous fashion throughout the macula. Our analysis approach uses a series of thin concentric rings spreading from the fovea towards the periphery with a width between 1 pixel and 28 pixels. By plotting the FD% calculated in each ring versus the distance from the fovea we obtained a detailed profile of the topographic distribution of the flow deficits in the CC. This curve resembles a sinusoid with irregular peaks and cycles. The shape of the curve is not surprising when one considers the structure of the CC, which is a dense net of capillaries. Of course, while a regular mesh would generate a more “regular” curve (Fig 4, box A), the relatively irregular CC mesh, together with the limitations of the methodologies used (e.g. resolution, magnification), yield a more irregular curve for the OCTA image (Fig 4, box C).

OCTA has already been shown to generate images which are consistent with histology [10–12] and our method seems to support this contention mathematically. Obtaining a continuous function of the topographical distribution of the flow deficits in the *en-face* angiograms allowed us to verify the association between the FD% and the distance from the fovea. This association is statistically significant and should be taken into consideration when two different regions of the CC are compared. In a population of subjects between 21 and 80 years, increasing the distance from the fovea leads to a physiological decrease of FD% which, according to our results, can be estimated to be approximately 0.8% for every 500 μm in the 3 x 3 mm and 0.25% for every 500 μm in the 6 x 6 mm.

**Fig 5:**
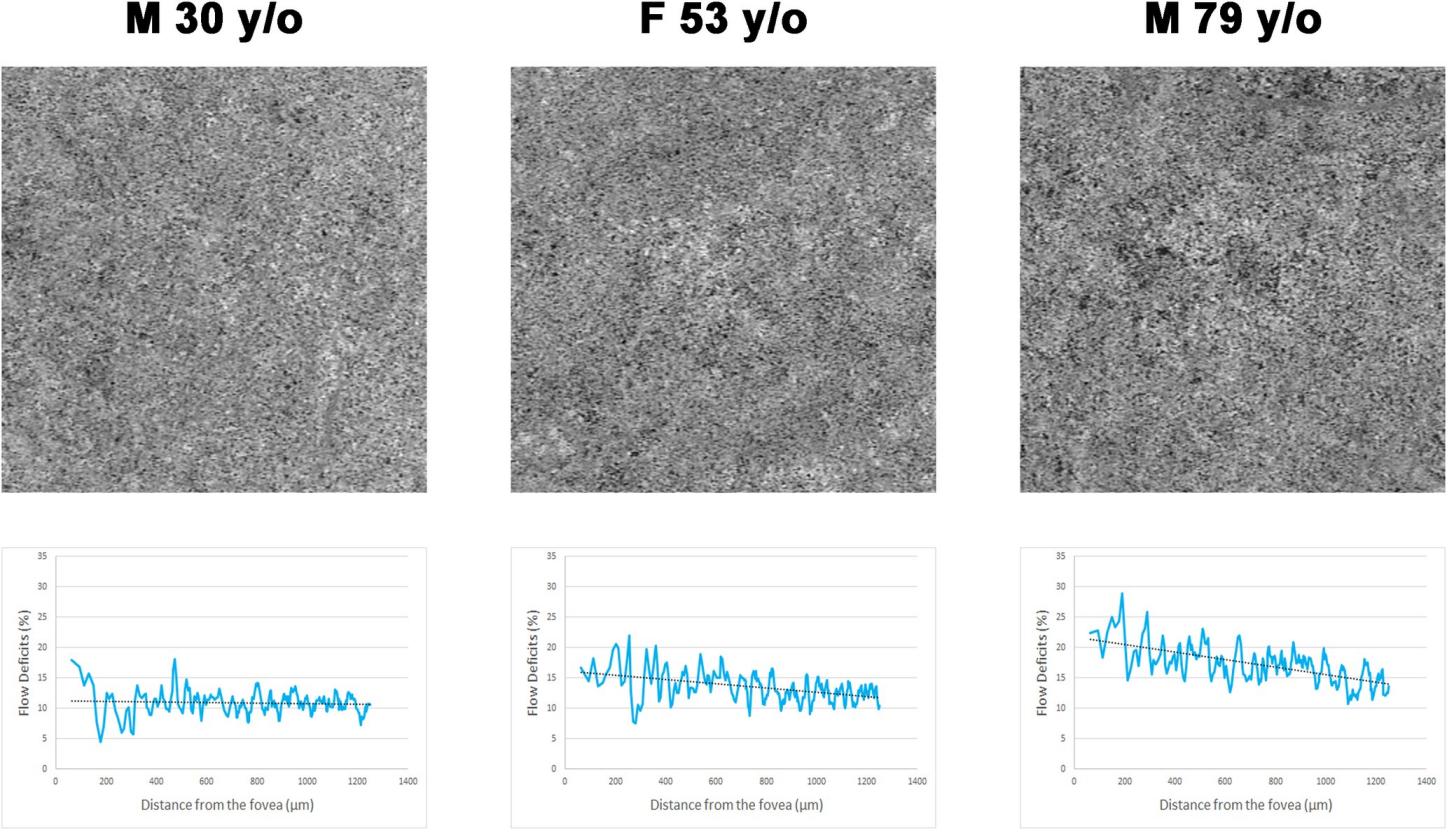
Examples of choriocapillaris *en face* optical coherence tomography angiograms from three healthy subjects at different ages. A qualitative evaluation of the angiograms (first row) shows an increase in flow deficits in the oldest subject compared to the younger examples. This qualitative observation is confirmed by the application of the quantitative method developed in this study (second row). The three curves generated by plotting the distance from the fovea (x-axis) against the percentage of flow deficits (y-axis) (solid blue lines) and their respective linear trendlines (dotted black lines) clearly shows a more flow deficits in the older subjects, especially closer to the foveal center.

Furthermore, we demonstrated that this increase is age-dependent: in older subjects, the impact of the distance from the fovea on FD% is greater, with a decrease of 1.4% and 0.8% for every 500 μm from the foveal center, in the 3 x 3 mm and 6 x 6 mm, respectively, for subjects between 61 and 80 years of age. These results, together with the analysis of the intercepts and slopes of the trendlines for the subjects, confirms that older individuals tend to have more flow deficits, and this age-dependent difference is greatest closer to the foveal center (Fig 5). It is important to note that this behavior was noted in both the 3x3mm and 6x6mm scan patterns, despite differences in resolution between the scan patterns.

It has already been demonstrated that the increasing age leads to a generalized reduction in the vessel density in all retinal vascular layers. The physiologic mechanism for this is not known, in particular for why this effect appears more pronounced centrally. Ramrattan et al. demonstrated in histologic samples that CC density decreases with age together with a significant increase in Bruch’s membrane (BM) thickness [21]. Whether these changes are uniform within the macula, however, was not specified and, to the best of our knowledge, never been investigated. An inhomogeneous thickening of BM, if more prominent under the fovea, could lead to an increased signal drop off on OCTA at the level of the CC, hence to an overestimation of the flow deficits in this zone.

Alternatively, it is possible that the CC under the fovea could be subjected to more stress to “recycle” the metabolites produced by the overlying RPE and photoreceptors, whose number/density is higher in this area. The foveal RPE cells are more tightly packed being smaller in diameter and taller compared to surrounding regions. This higher level of stress may be toxic for the CC leading to a faster impairment of the flow under the fovea with ageing. It is possible that after a “critical point”, the CC impairment under the fovea could lead to progressive pathological alterations at the level of the RPE (e.g. due to hypoxia and accumulation of metabolic debris) which may explaining the preference of some pathologies (e.g. age-related macular degeneration) to feature more pathology (e.g. regular drusen, macular neovascularization, or atrophy) near the center. If this hypothesis could be validated in future studies, it is possible that the monitoring of CC flow as an individual ages could prove to be useful tool to identify patients at risk for reaching this “critical tipping point”. Regardless, these regional differences in the CC that are accentuated by age must be taken into account in any studies comparing potential CC abnormalities in different parts of the macula. To address these regional differences, in a recent study evaluating CC flow deficits surround geographic atrophy lesions,[16] we compared regions at equal distances from the foveal center.

Our study is not without limitations, including a relatively small sample size. However, our study also has several strengths including its prospective design, the use of SS-OCTA, the use of two graders and repeat scan acquisitions to evaluate repeatability, and the use of two separate scan patterns (3x3 mm and 6x6 mm) which are the ones most commonly used in present CC studies in the literature. Considering the two angiograms had the same dimension (1024x1024 pixels), each ring of the grid covered a wider area when applied to the 6x6 mm scans compared to the 3x3 mm ones. This could potentially lead to a lower sensitivity in detecting small difference in flow deficits across the grid when using the wider pattern. However, the overall distribution of the flow deficits and their trend with respect to distance from the fovea, which are the main parameters of relevance in this study, were not significantly impacted by this. In summary, our study demonstrates that the distribution of flow deficits in the CC angiograms is regionally dependent and that aging seems to contribute to an increase of flow deficits, particularly as one approaches the fovea. These findings should be taken into account when assessing the CC in the setting of disease, particularly when studies are evaluating different macular regions or including subjects of varying ages.

## References

[1] Spaide RF, Fujimoto JG, Waheed NK, Sadda SR, Staurenghi G. Optical coherence tomography angiography. Prog Retin Eye Res. 2018;64: 1–55. 10.1016/j.preteyeres.2017.11.003 29229445 10.1016/j.preteyeres.2017.11.003 PMC6404988

[2] Olver JM. Functional anatomy of the choroidal circulation: methyl methacrylate casting of human choroid. Eye Lond Engl. 1990;4 (Pt 2): 262–272. 10.1038/eye.1990.38 2379644 10.1038/eye.1990.38

[3] Sacconi R, Corbelli E, Carnevali A, Querques L, Bandello F, Querques G. OPTICAL COHERENCE TOMOGRAPHY ANGIOGRAPHY IN GEOGRAPHIC ATROPHY. Retina Phila Pa. 2017; 10.1097/IAE.0000000000001873 29016457 10.1097/IAE.0000000000001873

[4] Borrelli E, Uji A, Sarraf D, Sadda SR. Alterations in the Choriocapillaris in Intermediate Age-Related Macular Degeneration. Invest Ophthalmol Vis Sci. 2017;58: 4792–4798. 10.1167/iovs.17-22360 28973325 10.1167/iovs.17-22360

[5] Spaide RF. Choriocapillaris Flow Features Follow a Power Law Distribution: Implications for Characterization and Mechanisms of Disease Progression. Am J Ophthalmol. 2016;170: 58–67. 10.1016/j.ajo.2016.07.023 27496785 10.1016/j.ajo.2016.07.023

[6] Rochepeau C, Kodjikian L, Garcia M-A, Coulon C, Burillon C, Denis P, et al OCT-Angiography Quantitative Assessment of Choriocapillaris Blood Flow in Central Serous Chorioretinopathy. Am J Ophthalmol. 2018; 10.1016/j.ajo.2018.07.004 30053475 10.1016/j.ajo.2018.07.004

[7] Spaide RF, Fujimoto JG, Waheed NK. IMAGE ARTIFACTS IN OPTICAL COHERENCE TOMOGRAPHY ANGIOGRAPHY. Retina Phila Pa. 2015;35: 2163–2180. 10.1097/IAE.0000000000000765 26428607 10.1097/IAE.0000000000000765 PMC4712934

[8] Choi W, Moult EM, Waheed NK, Adhi M, Lee B, Lu CD, et al Ultrahigh Speed Swept Source OCT Angiography in Non-Exudative Age-Related Macular Degeneration with Geographic Atrophy. Ophthalmology. 2015;122: 2532–2544. 10.1016/j.ophtha.2015.08.029 26481819 10.1016/j.ophtha.2015.08.029 PMC4658257

[9] Moult E, Choi W, Waheed NK, Adhi M, Lee B, Lu CD, et al Ultrahigh-Speed Swept-Source OCT Angiography in Exudative AMD. Ophthalmic Surg Lasers Imaging Retina. 2014;45: 496–505. 10.3928/23258160-20141118-03 25423628 10.3928/23258160-20141118-03 PMC4712918

[10] Zhang Q, Zheng F, Motulsky EH, Gregori G, Chu Z, Chen C-L, et al A Novel Strategy for Quantifying Choriocapillaris Flow Voids Using Swept-Source OCT Angiography. Invest Ophthalmol Vis Sci. 2018;59: 203–211. 10.1167/iovs.17-22953 29340648 10.1167/iovs.17-22953 PMC5770182

[11] Choi W, Mohler KJ, Potsaid B, Lu CD, Liu JJ, Jayaraman V, et al Choriocapillaris and choroidal microvasculature imaging with ultrahigh speed OCT angiography. PloS One. 2013;8: e81499 10.1371/journal.pone.0081499 24349078 10.1371/journal.pone.0081499 PMC3859478

[12] Gorczynska I, Migacz JV, Jonnal R, Zawadzki RJ, Poddar R, Werner JS. Imaging of the human choroid with a 1.7 MHz A-scan rate FDML swept source OCT system. Opthalmic Technologies XXVII. SPIE; 2017 p. 1004510 10.1117/12.2251704

[13] Borrelli E, Sarraf D, Freund KB, Sadda SR. OCT angiography and evaluation of the choroid and choroidal vascular disorders. Prog Retin Eye Res. 2018; 10.1016/j.preteyeres.2018.07.002 30059755 10.1016/j.preteyeres.2018.07.002

[14] Ouyang Y, Heussen FM, Mokwa N, Walsh AC, Durbin MK, Keane PA, et al Spatial distribution of posterior pole choroidal thickness by spectral domain optical coherence tomography. Invest Ophthalmol Vis Sci. 2011;52: 7019–7026. 10.1167/iovs.11-8046 21810980 10.1167/iovs.11-8046 PMC3176017

[15] Borrelli E, Shi Y, Uji A, Balasubramanian S, Nassisi M, Sarraf D, et al Topographical Analysis of the Choriocapillaris in Intermediate Age-related Macular Degeneration. Am J Ophthalmol. 2018; 10.1016/j.ajo.2018.08.014 30118688

[16] Nassisi M, Shi Y, Fan W, Borrelli E, Uji A, Ip MS, et al Choriocapillaris impairment around the atrophic lesions in patients with geographic atrophy: a swept-source optical coherence tomography angiography study. Br J Ophthalmol. 2018; 10.1136/bjophthalmol-2018-312643 30131381 10.1136/bjophthalmol-2018-312643

[17] Uji A, Balasubramanian S, Lei J, Baghdasaryan E, Al-Sheikh M, Sadda SR. Impact of Multiple En Face Image Averaging on Quantitative Assessment from Optical Coherence Tomography Angiography Images. Ophthalmology. 2017; 10.1016/j.ophtha.2017.02.006 28318637 10.1016/j.ophtha.2017.02.006

[18] Schneider CA, Rasband WS, Eliceiri KW. NIH Image to ImageJ: 25 years of image analysis. Nat Methods. 2012;9: 671–5. 22930834 10.1038/nmeth.2089 PMC5554542

[19] Uji A, Balasubramanian S, Lei J, Baghdasaryan E, Al-Sheikh M, Sadda SR. Choriocapillaris Imaging Using Multiple En Face Optical Coherence Tomography Angiography Image Averaging. JAMA Ophthalmol. 2017; 10.1001/jamaophthalmol.2017.3904 28983552 10.1001/jamaophthalmol.2017.3904 PMC5710392

[20] Spaide RF. CHORIOCAPILLARIS SIGNAL VOIDS IN MATERNALLY INHERITED DIABETES AND DEAFNESS AND IN PSEUDOXANTHOMA ELASTICUM. Retina. 2017; 1 10.1097/IAE.0000000000001497 28092344 10.1097/IAE.0000000000001497

[21] Ramrattan RS, van der Schaft TL, Mooy CM, de Bruijn WC, Mulder PG, de Jong PT. Morphometric analysis of Bruch’s membrane, the choriocapillaris, and the choroid in aging. Invest Ophthalmol Vis Sci. 1994;35: 2857–2864. 8188481

